# Supplementary material for: Therapeutic Exercise Prescription for Overhead Athletes with Shoulder Impingement Syndrome: A Systematic Review and CERT Analysis
Source: J Clin Med. 2025 Feb 28;14(5):1657. doi: 10.3390/jcm14051657 (PMC11901016; doi:10.3390/jcm14051657)
Supplement: Supplementary file 1 [file jcm-14-01657-s001.zip › Table S1_Search strategy.pdf]

Table S1. Search strategy

PubMed

#1 (((shoulder[Title] OR shoulders[Title] OR "shoulder pain"[Title/abstract] OR "shoulder impingement"[Title/abstract] OR "shoulder instability" [Title/abstract]) NOT (rotator cuff tendinopathy [Title] OR osteoarthritis[Title] OR arthroplast*[Title] OR fractur*[Title] OR "total shoulder replacement"[Title]])

#2 ((physical therap*[Title/Abstract] OR physiotherap*[Title/Abstract] OR exercise[Title/Abstract] OR training[Title/abstract] OR "manual therapy"[Title/Abstract] OR mobilization[Title/Abstract] OR mobilisation[Title/Abstract] OR conservative[Title/Abstract] OR "non operative"[Title/Abstract] OR "non surgical"[Title/Abstract] OR "patient education"[Title/Abstract] OR "Physical Therapy Modalities"[Majr]))))

#3 (Crossfit [Title] OR weightlifting[Title] OR high intensity interval training[Title] OR overhead athletes[Title] OR overhead lifting[Title] OR Crossfitters[Title])

4 #1 AND #2 AND #3

CINAHL

#1 TI (shoulder OR shoulders OR "shoulder pain"OR "shoulder impingement"OR "shoulder instability")

#2 TI (physical therap* OR physiotherap* OR exercise OR training OR "manual therapy" OR mobilization OR mobilisation OR conservative OR "non operative" OR "non surgical" OR "patient education" OR "Physical Therapy Modalities")

#3 TI (Crossfit OR weightlifting OR high intensity interval training OR overhead athletes OR overhead lifting OR Crossfitters)

#4 TI (rotator cuff tendinopathy OR osteoarthritis OR arthroplast* OR fractur* OR "total shoulder replacement")

#5 #1 AND #2 AND #3 NOT #4

SportDiscuss

#1 TI (shoulder OR shoulders OR "shoulder pain"OR "shoulder impingement"OR "shoulder instability")

#2 TI (physical therap* OR physiotherap* OR exercise OR training OR "manual therapy" OR mobilization OR mobilisation OR conservative OR "non operative" OR "non surgical" OR "patient education" OR "Physical Therapy Modalities")

#3 TI (Crossfit OR weightlifting OR high intensity interval training OR overhead athletes OR overhead lifting OR Crossfitters)

#4 TI (rotator cuff tendinopathy OR osteoarthritis OR arthroplast* OR fractur* OR "total shoulder replacement")

#5 #1 AND #2 AND #3 NOT #4

WOS

#1 TS=(shoulder OR "shoulder pain" OR "shoulder impingement" OR "shoulder instability")

#2 TS=(”rotator cuff tendinopathy” OR osteoarthritis OR arthroplast* OR fractur* OR "total shoulder replacement")

#3 TS=(”physical therap*” OR physiotherap* OR exercise OR training OR "manual therapy" OR mobilization OR mobilisation OR conservative OR "non operative" OR "non surgical" OR "patient education" OR "Physical Therapy Modalities")

#4 TS=(Crossfit* OR weightlifting OR ”high intensity interval training” OR ”overhead athletes” OR ”overhead lifting”)

#5 #1 NOT #2 AND #3 AND #4

Cochrane

#1 (shoulder* OR "shoulder pain" OR "shoulder impingement" OR "shoulder instability") in Title Abstract Keyword

#2 (”rotator cuff tendinopathy” OR osteoarthritis OR arthroplast* OR fractur* OR "total shoulder replacement") in Title Abstract Keyword

#3 (”physical therap*” OR physiotherap* OR exercise OR training OR "manual therapy" OR mobilization OR mobilisation OR conservative OR "non operative" OR "non surgical" OR "patient education" OR "Physical Therapy Modalities") in Title Abstract Keyword

#4 (Crossfit* OR weightlifting OR ”high intensity interval training” OR ”overhead athletes” OR ”overhead lifting” OR crossfitter*) in Title Abstract Keyword - (Word variations have been searched

#5 #1 NOT #2 AND #3 AND #4
